# Characteristics of antrochoanal polyps in the pediatric age group

**DOI:** 10.4103/1817-1737.53353

**Published:** 2009

**Authors:** Khalid A. Al-Mazrou, Manal Bukhari, Abdurhman I Al-Fayez

**Affiliations:** *Department of ORL, College of Medicine, King Saud University, Riyadh, Saudi Arabia*; 1 *ENT Division, College of Medicine, King Saud Bin A/Aziz University for Health Sciences, Riyadh, Saudi Arabia*

**Keywords:** Adults, antrochoanal polyp, children, pathology, surgery

## Abstract

**OBJECTIVES:**

To evaluate and compare the clinical and the pathological characteristics of antrochoanal polyps (ACPS) in adults and children.

**MATERIALS AND METHODS:**

Medical records of 35 patients (19 children, 16 adults) operated upon for ACPS between 1995 and 2005 at an academic tertiary center were reviewed retrospectively. Demographic characteristics, clinical presentation, surgical management, histological findings and recurrence rate were compared.

**RESULTS:**

Of the 35 patients, 19 (54%) were children (mean age, 12.6 years) and 16 (46%) were adults (mean age, 31.4 years). Nasal obstruction was the most common presenting symptom in both groups. The incidence of snoring and/or obstructive sleep apnea was statistically significant, more common among the pediatric age group as compared to the adult group (*P* =.001). Epistaxis was also found to be more common among the pediatric age group (*P* =.027), while sinusitis was noted to be significantly more common among the adult group (*P* =.019). Transnasal endoscopic removal of ACPS was performed in 12 (63.1%) children and 11 (68.7%) adults. A combined open/endoscopic approach was required in 36.9% of children and 31.3% of adults. On histologic examination, allergic ACPS (the mucosal surface is respiratory epithelium, no mucus glands, abundant eosinophils) was more common than inflammatory ACPS (the mucosal surface is respiratory epithelium, no mucus glands, abundant neutrophils) in children (2.8:1) as compared to adults (0.8:1) (*P* =.045). All of our patients were followed with endoscopic examination for a period ranging from 9 to 42 months (mean, 24 months). Recurrence of ACPS was identified in 2 children and 1 adult.

**CONCLUSION:**

Antrochoanal polyps are a rare clinical entity. Children have unique clinical and pathological features as compared to adults. Endoscopic excision is safe and effective in the pediatric age group and has the capability to ensure complete removal and lower recurrence rate.

Antrochoanal polyps (ACPS) were first described in a case report by Paefyn in 1713. Killian, however, is widely credited with the first detailed description of this entity in 1906.[1] ACPS are inflammatory polyps arising from the mucosa of the maxillary sinus and extending through the maxillary ostium into the nose. They represent approximately 4% to 6% of all nasal polyps in the general population.[2,3] In children, this number appears to be closer to 33% of all nasal polyps in the Pediatric population.[4]

Although some have proposed that ACPS develop as a complication of chronic inflammatory antral disease, in their review of ACPS in the pediatric population, Chen and his colleagues reported that 50% of the patients in their series had allergic diatheses.[5–8]

Although there are a few cases of bilateral ACPS presented in literature, ACPS are almost always unilateral. Nasal obstruction and drainage are the most common presenting symptoms; but in severe cases the presentation may be more dramatic, with symptoms of epistaxis, dyspnea, dysphagia and weight loss.[9,10]

Historically, treatment of involved avulsion of the polyp is often combined with a Caldwell-Luc procedure and curettage of the maxillary sinus mucosa. The advent of endoscopes and their use in nasal and sinus surgery has changed the management of this condition. We present our series of 19 children with antrochoanal polyps treated over a 10-year period, with attention to the clinical presentation, physical examination, radiological findings, surgical management, pathological features and recurrence rates. This group was compared to 16 adults treated for the same condition during the same period.

## Materials and Methods

A retrospective review of the patient database of the Department of Otolaryngology at King Abdulaziz University Hospital was performed for all patients treated for antrochoanal polyps.

The medical records were scrutinized, and the demographics, clinical presentation, treatment, histology and outcomes were recorded. The study population was then subdivided into pediatric (≤18 years of age) and adult (>18 years of age) groups for statistical analysis. The groups were matched in all aspects except age.

All patients underwent a complete history, head and neck examination, nasal endoscopy and computed tomography. One of the following two treatment strategies was applied: (1) endoscopic removal or (2) combined transantral and endoscopic approach.

The procedures were done under general anesthesia; the patients were placed in supine position with head slightly elevated. Initially, the uncinate process was removed with the pediatric back-biting forceps. The pedicle of the polyp was then transected with either a through-cutting forceps or the microdebrider. The nasal part of the polyp was removed transorally or transnasally, depending on the size. The antral portion of the polyp was removed through the natural maxillary ostium, which was wide enough in most of the cases; but widening of the ostium was needed in a few cases and was done using backward-cutting forceps to obtain good surgical view of the maxillary sinus. Any residual disease was removed with the help of 30- and 70-degree endoscopes and long forceps to access the most anterior inferior portion of the maxillary sinuses.

Additional access was obtained through the canine fossa in cases where the antrochoanal polyp was attached by a broad base to the maxillary sinus mucosa or when it was difficult to access endoscopically.

Statistical analysis was performed with SPSS v12.0 statistical software, and a two-tailed *P* value less than 0.05 was considered significant. Pearson's Chi-square test, Fisher exact test and the student *t* -test were used to compare the two groups.

## Results

Thirty-five patients treated for ACPS between January 1995 and December 2005 were identified. There were 19 (54.2%) children and 16 (45.8%) adults. In the pediatric group, there were 9 males and 10 females; the mean age was 12.6 years, with a range of 5 to 18 years. In the adult group, there were 11 males and 5 females; the age range was 20 to 57 years. No underlying cystic fibrosis or immotile cilia syndrome was present in either group.

**Figure 1:**
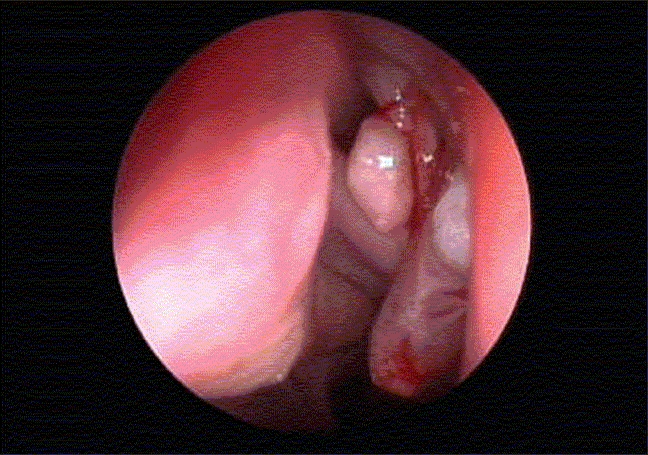
An endoscopic view of the left nasal cavity showing two antrochoanal polyps

The most frequent presenting complaint in both groups was unilateral nasal obstruction, followed by nasal discharge. The antrochoanal polyp was on the left side in 21 (60%) patients; while in rest (40%) of the patients, it was on the right side, with only 1 (2.8%) child having bilateral ACPS and another child having two coming from one side [Figure 1]. The nasal side of the ACPS did not differ significantly between the two groups.

We identified 11 patients (11/19,57.8%) complaining of snoring and/or obstructive sleep apnea (OSA) in the pediatric group. In the adults group, 2 (12.5%) patients showed features of OSA. The difference in the incidence of OSA between the pediatric and adult groups was statistically significant (*P* <.05). Headache was found in 15.7% of the pediatric patients and 37.5% of the adult patients, respectively, which did not show a statistically significant difference [Table 1].

**Table 1:** Clinical characteristics of antrochoanal polyps

Characteristics	Children no. (%)	Adult no. (%)	*P* value
Sex (M:F)	0.9:1	1.7:1	
Nasal obstruction			
Unilateral	18 (94.7)	15 (93)	
Bilateral	1 (5.2)	1 (6.2)	
Nasal drainage	15 (78.9)	11 (68.7)	0.336
Obstructive sleep apnea	11 (58)	2 (12.5)	0.001
Epistaxis	7 (37)	1 (6)	0.027
Headaches	3 (15)	6 (37.5)	0.176
Sinusitis	5 (26)	11 (96)	.0019
Allergy	7 (37)	7 (44)	0.777
Histopathology	2.8:1	0.8:1	0.045
(Allergic: Inflammatory)			
Recurrence	2 (11)	1 (6)	0.623

Physical examination in all patients showed intranasal polyp arising from the middle meatus and extending to the nasopharynx, in addition to endoscopic features of chronic sinusitis.

Computed tomography (CT) of the sinuses revealed maxillary sinus opacification in all cases. However, in the pediatric group, unilateral maxillary sinus involvement was found in 84.2% of the patients, whereas contralateral maxillary sinus involvement was found in 15.7% of the patients. In the adult group, the maxillary sinus involvement was both unilateral and contralateral, in 68.7% and 31.2% of the patients, respectively.

Transnasal endoscopic removal of ACPS was performed in 12 (63.1%) pediatric patients and 11 (68.7%) adult patients. The combined approach was applied in 36.9% of the children and 31.3% of the adults.

**Figure 2:**
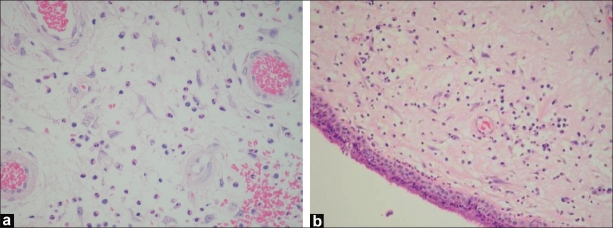
Microscopic view of two antrochoanal polyps. (a) Allergic type with abundant eosinophils; (b) Inflammatory type with abundant neutrophils

On histopathology, allergic polyps were found to be more common than inflammatory polyps among children (2.8:1); while in adults, inflammatory polyps were more common than allergic polyps (0.8:1). The difference is statistically significant (*P* <.05) [Figure 2].

Recurrence of ACPS was identified in 2 pediatric patients and 1 adult patient. All of the patients were followed up with endoscopic examination for a period ranging from 9 to 42 months, with a mean of 24 months.

## Discussion

Antrochoanal polyps in children are uncommon but occur at a higher rate than in the adult population. To our knowledge, this is the only comparative study of antrochoanal polyps in adults and children in the English language literature. ACPS are reported to represent one third of all nasal polyps in children.[4] In our study, 54.4% of all cases were among children. Although we noted a slight male preponderance (male-female ratio, 1.2:1), others have noted a female preponderance.[8] Left-sided ACPS were more common than right-sided (60% versus 40%) polyps, as reported by others as well.[4]

The most common symptom in both groups was unilateral nasal obstruction, followed by rhinorrhea (74%). Epistaxis (37%) and snoring and obstructive sleep apnea (OSA) (58%) were more common in children than in adults. Epistaxis is an unusual manifestation of ACPS in such cases. Angiofibroma must be excluded in pediatric male patients; and malignancy, in adult patients. Other reviewers[11,12] found that OSA was uncommon as an initial presentation of ACPS among children, which is in contrast to our findings. Three explanations were given for this issue. First, higher mean age of children with the that combined with significantly larger size of nasopharynx compared to younger age with adenoid enlargement. Secondly, there is the incomplete blockage of the nose by the ACPS. Third explanation is that ACPS in children present less often with apnea than in adults.[12]

Chronic sinusitis has been implicated as a cause of ACPS, but this has not been proven conclusively.[13] In our study, 11 (96%) patients from the adults group had chronic sinusitis; while in the pediatric group, only 5 (26%) patients had chronic sinusitis. This is consistent with the findings of others,[14] who reported that 50% of patients had concomitant sinusitis. Intractable chronic sinusitis is more difficult to treat in patients with ACPS.[15]

Computed tomography is the radiological evaluation of choice for evaluation of antrochoanal polyps. Classically it demonstrates a hypodense mass arising from an opacified maxillary sinus extending through the middle meatus into the nasal cavity. As the polyp enlarges, it may extend posteriorly toward the choana into the nasopharynx.[16] In our series, CT scanning revealed bilateral maxillary sinus involvement in 6 cases, equally divided between the adult and pediatric populations. This involvement was documented as well on nasal endoscopy examination. This highlighted the importance of CT scan for diagnostic and preoperative planning.

Surgery is the usual treatment for symptomatic antrochoanal polyps. Historically, surgical therapy has involved 2 different procedures—simple avulsion of the polyp or Caldwell-Luc procedure. Simple avulsion alone of the polyp carries a high recurrent rate.[3,17] The Caldwell-Luc procedure offers excellent exposure and ensures complete removal of the antral part of the polyp.[4] It however carries significant risks to the developing teeth and the bone growth centers of the maxilla, as well as risk of facial hypesthesia. Long recovery times are also common.[18]

More recently, endoscopic excision has become the procedure of choice because of its shorter recovery time and fewer side effects.[19,20–22] Complete removal of the maxillary portion of the polyp is necessary to decrease recurrence rates. Kamal suggested endoscopic excision of the antral polyp through a middle meatal antrostomy and reported no recurrence in his series of 22 patients using this approach.[22] In our study, 23 patients (12 children and 11 adults) underwent endoscopic removal of the ACPS.

Microdebriders have been reported to facilitate removal and reduce the limitation of the conventional endoscopic approach.[23,24] When there is broad-based attachment to the anterior or inferior walls of maxillary sinus, a transcanine fossa approach is usually combined with the endoscopic one for complete removal.[23,25] In our series, 3 patients (2 children and 1 adult) had polyps removed by the combined approach after failure of the endoscopic approach; there have been no recurrences in those patients in 24 months. We now reserve this approach for cases where the antral part is not accessible or there is recurrence after endoscopic excision.

Histologically, there is essentially no difference between antrochoanal polyps and inflammatory nasal polyps. Significant association between ACPS and allergy status was found in 33 cases, confirmed by history and by *in vivo/in vitro* diagnostic tests.[19] Histopathological specimens revealed that allergic polyps (abundant eosinophils) were significantly more common than inflammatory polyps (abundant neutrophils) among children (2.8:1). While in adults, inflammatory polyps were more common than allergic polyps (0.8:1). In our study, 40% of our patients had symptoms of seasonal allergic rhinitis, but no allergy testing was done.

The recurrence rate in endoscopic surgical treatment of the ACPS is usually low.[22] Our series corroborated these findings.

## Conclusion

Antrochoanal polyps are rare in children and adults. This is the first series to compare ACPS in adults and children. In contrast to adults, allergic polyps were more common than inflammatory polyps among children. Endoscopic approach is safe and effective in the treatment of ACPS in both children and adults and has the capability to ensure complete removal and lower recurrence rate.
